# iPMI: Machine Learning-Aided Identification of Parametrial Invasion in Women with Early-Stage Cervical Cancer

**DOI:** 10.3390/diagnostics11081454

**Published:** 2021-08-12

**Authors:** Phasit Charoenkwan, Watshara Shoombuatong, Chalaithorn Nantasupha, Tanarat Muangmool, Prapaporn Suprasert, Kittipat Charoenkwan

**Affiliations:** 1 College of Arts, Media and Technology, Chiang Mai University, Chiang Mai 50200, Thailand; phasit.c@cmu.ac.th; 2 Center of Data Mining and Biomedical Informatics, Faculty of Medical Technology, Mahidol University, Bangkok 73170, Thailand; watshara.sho@mahidol.ac.th; 3 Division of Gynecologic Oncology, Department of Obstetrics and Gynecology, Faculty of Medicine, Chiang Mai University, Chiang Mai 50200, Thailand; chalaithorn.n@cmu.ac.th (C.N.); tanarat.m@cmu.ac.th (T.M.); prapaporn.su@cmu.ac.th (P.S.)

**Keywords:** cervical cancer, parametrial invasion, health informatics, machine learning, random forest

## Abstract

Radical hysterectomy is a recommended treatment for early-stage cervical cancer. However, the procedure is associated with significant morbidities resulting from the removal of the parametrium. Parametrial cancer invasion (PMI) is found in a minority of patients but the efficient system used to predict it is lacking. In this study, we develop a novel machine learning (ML)-based predictive model based on a random forest model (called iPMI) for the practical identification of PMI in women. Data of 1112 stage IA-IIA cervical cancer patients who underwent primary surgery were collected and considered as the training dataset, while data from an independent cohort of 116 consecutive patients were used as the independent test dataset. Based on these datasets, iPMI-Econ was then developed by using basic clinicopathological data available prior to surgery, while iPMI-Power was also introduced by adding pelvic node metastasis and uterine corpus invasion to the iPMI-Econ. Both 10-fold cross-validations and independent test results showed that iPMI-Power outperformed other well-known ML classifiers (e.g., logistic regression, decision tree, k-nearest neighbor, multi-layer perceptron, naive Bayes, support vector machine, and extreme gradient boosting). Upon comparison, it was found that iPMI-Power was effective and had a superior performance to other well-known ML classifiers in predicting PMI. It is anticipated that the proposed iPMI may serve as a cost-effective and rapid approach to guide important clinical decision-making.

## 1. Introduction

Cervical cancer is the fourth most common cancer in women following breast, colorectal, and lung cancers. It is also the fourth leading cause of death from cancer [1]. Cancer cells’ ability to invade surrounding tissues as well as metastasize to regional lymph nodes and distant organs is responsible for more than 90% of cancer-associated deaths [2]. Cervical cancer usually spreads in a stepwise fashion from primary cervical tumor to adjacent structures including the parametrium, vagina, urinary bladder, and rectum. The cancer cells can also metastasize to regional lymph nodes and distant sites [3]. 

For early-stage (FIGO stage IA2-IIA) cervical cancer, parametrial invasion (PMI) and pelvic node metastasis are associated with a higher risk of recurrence and poorer chances of survival [4,5,6]. Therefore, the primary surgical treatment for these patients usually includes a radical hysterectomy with the removal of the adjacent parametrium and a pelvic lymphadenectomy [7]. The treatment is generally effective with satisfactory survival outcome [8]. However, significant intraoperative complications such as excessive blood loss and injury to adjacent organs, as well as long-term morbidities including voiding dysfunction, lower gastrointestinal dysfunction, and sexual dysfunction, are frequently encountered [9,10,11,12,13,14]. These conditions mainly result from trauma to the pelvic blood vessels and autonomic nerves during parametrial resection [9,15]. In an attempt to minimize these long-term nerve-related morbidities, the nerve-sparing technique for radical hysterectomy has been adopted. However, significant postoperative morbidities are still observed [16]. In addition to radical surgery, if cancer metastasis in the parametrium/pelvic nodes, or involved surgical margins, are identified, adjuvant postoperative pelvic radiation with concurrent chemotherapy is indicated [17,18]. This would further increase the incidence of posttreatment morbidities in the patients who receive combined therapeutic modalities.

For these early-stage patients however, the reported incidence of PMI ranges from 5–25% [19,20]. Thus, the majority of patients undergo aggressive “radical” surgery unnecessarily. Therefore, the accurate prediction of PMI among patients with early-stage cervical cancer due to have surgery can facilitate the rapid identification of patients with a low risk of metastasis, for whom removal of parametria is not necessary. In this case, a radical hysterectomy could be replaced by the less aggressive simple hysterectomy. As a result, treatment-related complications could be significantly diminished. On the other hand, for those preoperatively classified as having a high risk of PMI, primary concurrent chemoradiation (CCRT) can be seriously considered with primary radical hysterectomy remaining as an alternative option. This particular approach provides good oncological outcomes with a substantial reduction in morbidity. However, the efficient system to predict PMI is currently insufficient.

In this study, we propose a novel machine learning (ML)-based predictive model called iPMI for the practical identification of PMI in women with early-stage cervical cancer who are candidates for primary radical surgery. This category of modeling technique is increasingly employed in cancer prognostic model development studies with highly reliable predictive performance [21,22,23]. To validate the effectiveness and robustness of the iPMI model developed by using the random forest (RF) method, we compared its predictive performance with those of conventional logistic regression (LR) and other widely used ML classifiers including decision tree (DT), k-nearest neighbor (kNN), multi-layer perceptron (MLP), naive Bayes (NB), support vector machine (SVM), and extreme gradient boosting (XGB).

## 2. Materials and Methods

### 2.1. Data Source and Study Population

Clinical and pathological data of 1112 patients with clinical FIGO stage IA-IIA cervical cancer who underwent primary radical hysterectomies and pelvic lymphadenectomies at our department from January 2003 to December 2016, were used as a training dataset. Specifically, patients with a tumor size >4 as well as those who received preoperative chemotherapy were excluded. To validate the effectiveness of the model, an independent test dataset of 116 consecutive women with FIGO stage IA-IIA cervical cancer treated at our hospital from January 2017 to July 2018, was established. The training and independent test datasets were obtained from the Division of Gynecologic Oncology database. Please note that this study was conducted under the approval of the Faculty of Medicine Research Ethics Committee (approval number OBG-2560-04901).

### 2.2. Outcome

The primary outcome was PMI, which was defined as microscopic pathological evidence of metastatic cancer to either unilateral or bilateral parametrial tissue or parametrial lymph nodes in the standard pathological assessment of radical hysterectomy specimens. At our institution, this information is usually available within one week following the primary surgery. We aimed to evaluate the association between PMI and its potential clinicopathological predicting factors. These factors included age, parity, human immunodeficiency virus (HIV) infection status, menopausal status, underlying diseases, previous abdominal surgery, prior conization, tumor size, tumor appearance (no gross lesions, exophytic, infiltrative, ulcerative, or mixed), stage (IA, IB1, IB2, IIA), histological type, histological grade, depth of cervical stromal invasion (inner third, middle third, outer third), lymph-vascular space invasion (LVSI), uterine metastasis, vaginal metastasis, vaginal margin status, adnexal metastasis, and pelvic lymph node metastasis.

### 2.3. Conventional Statistical Analysis

Association between individual clinicopathological factors and PMI was initially assessed by employing conventional statistical analysis. Herein, Fisher’s exact test was used to compare clinicopathological factors between PMI and non-PMI groups. The univariable analysis selected clinicopathological factors with *p*-value ≤0.10 for entering a multivariable LR model. The backward selection was applied in multivariable analysis to identify independent predicting factors for PMI. A *p*-value of ≤0.05 denoted a statistical significance.

### 2.4. Predictive Models

#### 2.4.1. Synthetic Minority Oversampling Technique

The number of patients in PMI group was relatively small compared to that of patients in non-PMI group, with a PMI to non-PMI ratio of 1:4.64. This may have affected the predictive ability of the model for accurately identifying the minority class (PMI group). Class imbalance was a problem arising in many practical applications and caused issues of bias during the learning and prediction process [24,25,26,27,28]. This problem may have decreased the prediction performance of computational predictors. Therefore, the sample rescaling-based method containing oversampling and undersampling approaches was proposed to alleviate the class imbalance problem and remove the biasness [25,26,29]. In general, the undersampling approach was used for eliminating some of the samples from the majority class (no PMI) while the oversampling approach was used for creating new samples from the minority class (PMI). In the present study, we employed the synthetic minority oversampling technique (SMOTE) for performing oversampling of PMI group to introduce its synthetic samples [29].

#### 2.4.2. Development of Preoperative Computational Models

With the aim of developing the model that was useful in real practice, the clinical and pathological factors that could be determined preoperatively were used as input variables for the RF model. The combination of age, parity, HIV infection status, menopausal status, underlying diseases, prior conization, tumor size, stage, and histological type was considered as baseline factors in the model. In addition, the impacts of pelvic node metastasis (pelvicme), uterine corpus invasion (utmet), and vaginal metastasis (vgmet) were examined for their potential in improving the model’s predictive performance. The RF model was an ensemble-based ML algorithm used to perform classification and regression tasks which was introduced by Breiman [30,31]. Until now, the RF model was widely used in various applications [26,32,33,34,35,36,37,38]. Like many other ensemble ML methods, this method was developed by growing a number of weak classification and regression tree (CART) classifiers for improving the predictive performances of the CART classifiers [31,39]. The RF model employed the concepts of bagging and random feature selection. We obtained the prediction result of the classification task by using a voting method from a number of CART classifiers. In regression, a final prediction was the average of many prediction results of many CART classifiers. To improve the prediction performance of the RF model, two parameters, ntree (the number of tree used for constructing the RF classifier) and mtry (the number of random candidate features), were considered with a cross-validation technique. The search space of ntree were in (20, 50, 100, 200, 500).

#### 2.4.3. SHAP Analysis

Recently, SHAP (SHapley Additive exPlanations) was developed for explaining the prediction results of any ML model [40]. This approach was based on game theory and employed an additive feature attribution method allowing users to establish an interpretable model. In SHAP approach, the importance for each predicting factor was ranked by the SHAP value. This value indicated the importance for the ith feature by comparing the different output among the model with and without the ith feature. The feature with the largest absolute SHAP value was of the most importance. Meanwhile, the feature with a high positive SHAP value had a positive impact on the output of ML model and vice versa. In the present study, we used the SHAP approach to determine the clinicopathological factors that are beneficial for PMI identification.

#### 2.4.4. Models’ Performance Evaluation

For the evaluation of the predictive model performance in the training dataset (cross-validation) and the independent testing dataset (independent test), the following four standard metrics in binary classification (PMIs and non-PMIs) were employed to assess discriminative ability of the proposed model:

(1) $$\mathrm{Ac}=\frac{\mathrm{TP}+\mathrm{TN}}{\left(\mathrm{TP}+\mathrm{TN}+\mathrm{FP}+\mathrm{FN}\right)}$$

(2) $$\mathrm{Sn}=\frac{\mathrm{TP}}{\left(\mathrm{TP}+\mathrm{FN}\right)}$$

(3) $$\mathrm{Sp}=\frac{\mathrm{TN}}{\left(\mathrm{TN}+\mathrm{FP}\right)}$$

(4) $$\mathrm{MCC}=\frac{\mathrm{TP}\times \mathrm{TN}-\mathrm{FP}\times \mathrm{FN}}{\sqrt{\left(\mathrm{TP}+\mathrm{FP}\right)\left(\mathrm{TP}+\mathrm{FN}\right)\left(\mathrm{TN}+\mathrm{FP}\right)\left(\mathrm{TN}+\mathrm{FN}\right)}}$$

where Ac, Sn, Sp and MCC are accuracy, sensitivity, specificity, and Matthews correlation coefficient, respectively. More details of these four standard metrics can be found in our previous studies [25,26,41,42,43,44,45]. Furthermore, the area under the receiver operating characteristic (ROC) curve was used to assess the predictive performance, where AUC values of 0.5 and 1 were indicative of random and perfect models, respectively.

## 3. Results

### 3.1. Patients’ Characteristics

Of 1112 patients, 171 patients (15.4%) had PMI. In conventional multivariable analysis using the LR method, only pelvic node metastasis, uterine corpus metastasis, tumor size ≥2 cm, vaginal metastasis, and menopause were significantly associated with PMI. Of note, adenocarcinoma histology was independently associated with a lower risk of PMI with the adjusted odds ratio of 0.49 (95% confidence interval 0.31–0.78) compared to squamous cell carcinoma histology.

Table 1 compares the clinicopathological factors between the training and the independent testing dataset. The prevalence of PMI was significantly higher in the testing dataset (*p* < 0.01). Additionally, the prevalence of vaginal metastasis was significantly higher in the testing dataset (*p* = 0.03). In addition, the prevalence of HIV positivity and previous abdominal surgery in the testing set was higher. All other factors appeared comparable between the two groups. 

### 3.2. Effect of Balanced and Imbalanced Datasets

**Figure 1:**
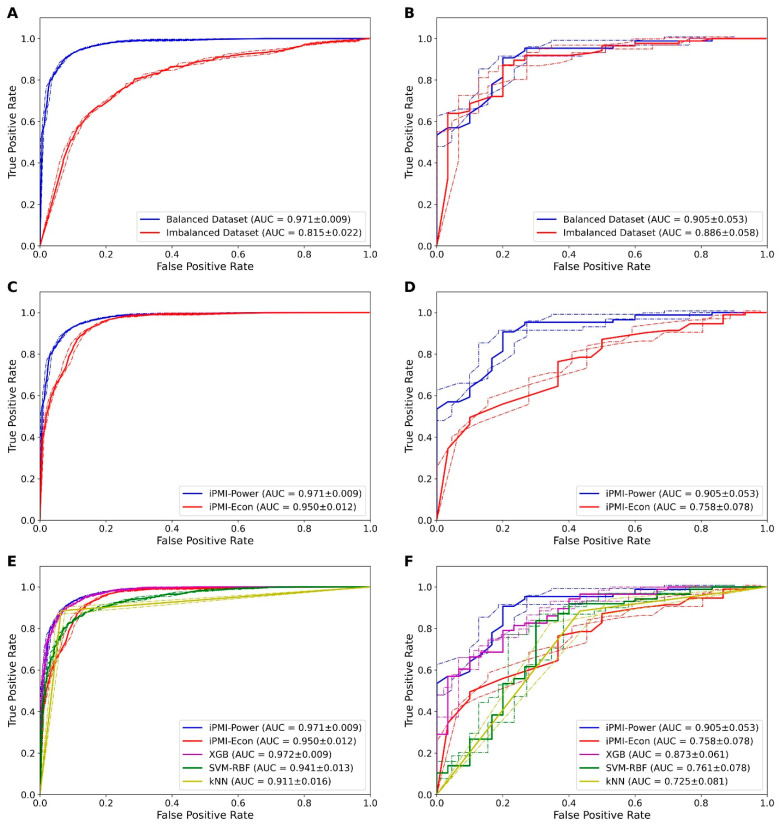
ROC curves of RF models trained using different feature sets based on the 10-fold cross-validation test (**A**,**C**,**E**) and independent test dataset (**B**,**D**,**F**): (**A**,**B**) illustrate comparison of RF models with and without the oversampling technique SMOTE; (**C**,**D**) illustrate comparison of iPMI-Power and iPMI-Econ, where iPMI-Power and iPMI-Econ are constructed by RF models trained using the combination of baseline+pelvicme+utmet and baseline factor, respectively; and (**E**,**F**) illustrate a comparison of the proposed models (i.e., iPMI-Power and iPMI-Econ) with kNN, SVM-RBF and XGB. The AUC values are expressed with 95% confidence interval.

To cope with class imbalance, we employed the SMOTE for performing the oversampling of the PMI group [29]. Herein, we conducted the performance comparison of RF models in conjunction with the preoperative clinicopathological or the baseline factor on balanced and imbalanced datasets. Figure 1A,B summarizes the 10-fold cross-validation in the training dataset and the independent test results in the testing cohort of RF models on imbalanced and balanced datasets. The RF model performing on the balanced dataset achieved a higher cross-validation AUC than the RF model performing on the imbalanced dataset. These results indicated that the performance of the RF model improved when the SMOTE oversampling technique was applied for adding samples to the PMI group. Therefore, we utilized the balanced dataset for further development of computational predictive models. It should be noted however, that the difference in the model performance between the balanced and the imbalanced datasets was less clear in the independent testing dataset.

### 3.3. Performance of Preoperative and Postoperative Clinicopathological Factors

In order to understand the contribution of each factor in PMI identification, a series of comparative experiments was carried out among various feature sets derived from combinations of clinicopathological factors (baseline, baseline+pelvicme, baseline+utmet, baseline+vgmet, baseline+pelvicme+utmet, baseline+pelvicme+vgmet, baseline+utmet+vgmet and baseline+pelvicme+utmet+vgmet). The prediction results of RF models trained using various combinations of clinicopathological factors based on the 10-fold cross-validation and independent tests are illustrated in Supplementary Table S1 and Table 2, respectively. We noticed that the best cross-validation Ac was achieved by using the combination of baseline+pelvicme+utmet+vgmet (0.918), the combination of baseline+pelvicme+vgmet (0.918), and the combination of baseline+pelvicme+utmet (0.915). For the independent test results, the combination of baseline+pelvicme+utmet provided the best Ac value of 0.862 with the clearly superior MCC of 0.618 and AUC of 0.905, compared to other feature sets. Moreover, as seen in Figure 1C,D, the combination of baseline+pelvicme+utmet had a superior performance compared to the baseline feature set, when considering cross-validation and independent test results. Altogether, the combination of baseline+pelvicme+utmet was the most beneficial combination for PMI identification. For the convenience of illustration, we refer to this method as the iPMI-Power. Meanwhile, the RF model trained with only the baseline factor provides the satisfactory Ac of 0.756. Therefore, this model was introduced as a simple approach (called the iPMI-Econ). Finally, in order to maximize the utility of the proposed model, we set up a publicly accessible web server at: http://camt.pythonanywhere.com/PMIPred.

### 3.4. Comparison of iPMI with Other ML Classifiers

To validate the effectiveness of the proposed models, we compared their predictive performances against well-known ML classifiers. Herein, we selected DT, LR, MLP, NB, XGB, and SVM models. In order to make a fair comparison, the DT, LR, MLP, NB, XGB, and SVM models were constructed based on the same feature set (the combination of baseline+pelvicme+utmet) using Scikit-Learn package [46]. This package has been successfully applied to various domains [25,41,42,43,44,45]. To demonstrate the comparative results clearly, we summarized the Ac, Sn, Sp, MCC and AUC values for iPMI-Power, iPMI-Econ and other ML classifiers assessed via 10-fold cross-validation (Table S2 and Figure 1E) and independent tests (Table 3 and Figure 1F). The iPMI-Power exhibited the best Ac, MCC, and AUC compared to other classifiers in both the 10-fold cross-validation and the independent tests. 

### 3.5. Analysis of Informative Clinicopathological Factors

**Figure 2:**
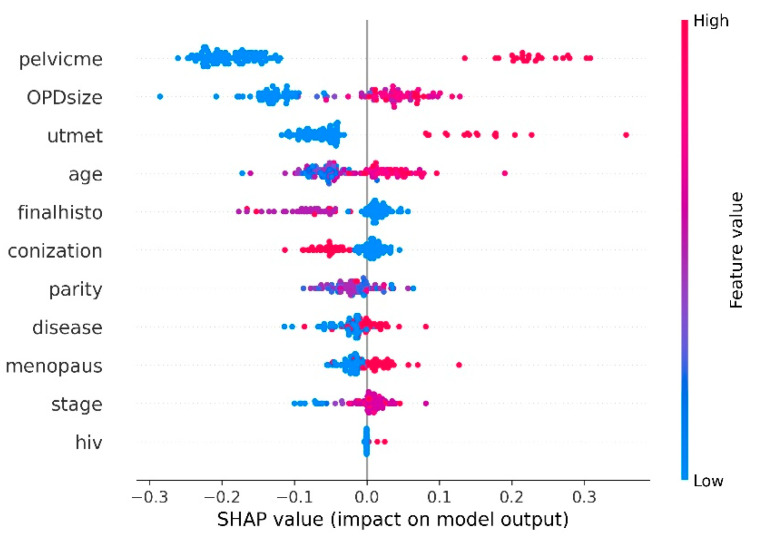
Clinicopathological factor analysis represents the relative importance for each factor obtained by averaging absolute SHAP values.

The SHAP approach provided information regarding the impact of individual predicting factors on the directionality of the output of the model. In this study, we passed the proposed iPMI-Power along with the balanced dataset to obtain the SHAP value for each clinicopathological factor. As shown in Figure 2, the five top-ranked important clinicopathological factors included pelvic node metastasis, tumor size (as measured at outpatient department), uterine corpus invasion, age, and histology.

## 4. Discussion

To establish an effective predictive model, we first collected the training and independent test datasets containing 1112 and 116 women, respectively, with FIGO stage IA-IIA cervical cancer treated at our hospital. Due to the class imbalance between PMIs and non-PMIs, the oversampling technique SMOTE was used to address the class imbalance problem as well as to remove bias. Based on the balanced dataset, iPMI-Econ was developed by using the RF model trained with the baseline clinicopathological factors that were generally recognized prior to surgery and included age, parity, HIV infection status, menopausal status, underlying diseases, prior conization, tumor size, stage, and histological type. To maximize the utility of the baseline clinicopathological factors, we effectively combined with pelvic node metastasis and uterine corpus metastasis to obtain iPMI-Power. Our empirical studies based on cross-validation and independent tests demonstrated the effectiveness of the iPMI-Power model by outperforming well-known ML classifiers, e.g., DT, LR, MLP, NB, XGB, and SVM. In the case of the iPMI-Econ model, however, its performance was worse than that of the well-known ML classifiers.

The necessity of parametrial removal by performing a radical hysterectomy for early-stage cervical cancer patients, especially those with FIGO stage IA2 to small IB1 disease, was challenged. In a recent meta-analysis addressing the impact of LVSI and pelvic node metastasis on PMI, the reported prevalence of PMI in early-stage cervical cancer varied from 0.6% to 32.5% in the 20 included studies [47]. Factors found to be associated with PMI included large tumor size, pelvic node metastasis, LVSI, deep cervical stromal invasion, histology, high tumor grade, uterine metastasis, and vaginal involvement. A subset of patients at very low risk for PMI (risk < 1%) were identified with various combinations of tumor sizes smaller than 2 cm, negative pelvic nodes, negative LVSI, and no more than inner third cervical stromal invasion [6,48,49,50,51,52]. However, apart from the tumor size, other factors incorporated in the proposed combinations could not be reliably determined before surgery. In addition, for the remaining early-stage patients, the reliable prediction of PMI was not attainable.

Landoni et al. examined the effects of simple extrafascial (class I) hysterectomy versus radical (class III) hysterectomy in 125 patients with stage IB1 and IIA cervical cancer, with a tumor size of ≤4 cm (class I 62 patients and class III 63 patients), in a randomized controlled trial [53]. Sixty-nine percent of the patients in the class I group and 55% of those in the class III group received adjuvant radiation (*p* = 0.11). Although recurrence rates were not statistically different; 24% in the class I group and 13% in the class III group (*p* = 0.11), it appeared worrisome for those who had a class I hysterectomy. Likewise, the overall five-year survival rate was 85% for the class I group and 95% for the class III group (*p* = 0.11). However, for patients with a tumor size of 3.1–4 cm, the authors noted a significant difference in 15-year overall survival between the two study groups; 74% in the class I group and 97% in the class III group (*p* = 0.03). Sia et al. recently reviewed the National Cancer Database regarding the uses and outcomes of a simple hysterectomy versus a radical hysterectomy for patients with stage IA2 and small IB1 (≤2 cm) [54]. Of 1530 women with stage IA2, 44.6% had a simple hysterectomy and for 3931 women with stage IB1, 35.3% had a simple hysterectomy. For women with stage IA2, no association between the type of hysterectomy and survival was identified; the hazard ratio (HR) of death was 0.70, with a 95% confidence interval (CI) 0.41–1.20. However, for patients with small stage IB1 disease, those who underwent a simple hysterectomy had a 55% increase in the risk of death (HR 1.55, 95% CI 1.18–2.03) compared with a radical hysterectomy. These findings suggest that without a more accurate system for PMI prediction, liberal modifications of surgical treatment for this particular group of patients could be potentially harmful.

Therefore, we aimed to develop a predictive model that could provide more accurate information about the risk of PMI for individual patients enabling the classification of patients based on risk. As the relationship between clinicopathological factors is frequently non-linear, it is difficult for the conventional statistical model to serve this task. A supervised machine learning model could effectively reduce bias and fit the data more appropriately. For the model to be useful in clinical decision making, e.g., performing a simple hysterectomy, proceeding with a radical hysterectomy, or switching to primary chemoradiation, we employed clinicopathological factors that could potentially be determined prior to surgery. Given the known association between cervical cancer and low socioeconomic status, we chose the factors that were accessible in low-resource settings. In addition, the measurement of these factors was practical and reasonably reliable.

We demonstrated that using combinations of both preoperative and postoperative clinicopathological factors afforded better prediction results than employing only baseline preoperative clinicopathological factors (Table 2). Additionally, the cross-validation and independent test results revealed that the combination of baseline+pelvicme outperformed the combinations of baseline+utmet and baseline+vgmet, indicating that pelvic node metastasis was more effective and robust in discriminating PMI from non-PMI than uterine corpus metastasis and vaginal metastasis. This finding was consistent with the findings from all previous reports on risk factors for PMI in early-stage cervical cancer [6,48,49,51]. Among the various combinations of predicting factors, the baseline+pelvicme+utmet combination offered the best performances in both the 10-fold cross validation of the training set and the independent dataset testing, leading to the proposed iPMI-Power model. The model outperformed other well-known ML classifiers. Of note, although the iPMI-Econ, which was a simpler and more economical model, delivered an impressive performance in the 10-fold cross validation of the training dataset, this could not be reproducible in the validation phase using the independent dataset.

The two versions of our model, the iPMI-Econ and the iPMI-Power, served the same purpose—predicting PMI in women with stage IA2-IIA cervical cancer. In the situation that PMI is predicted (high probability for PMI), primary concurrent chemoradiation should be seriously considered while a radical hysterectomy and a pelvic lymphadenectomy remains an alternative option. On the other hand, if no PMI is predicted (low probability for PMI), a simple hysterectomy could reasonably be proposed in place of a radical hysterectomy combined with a pelvic lymphadenectomy. It should be noted however, that the results of this study should be considered exploratory at this stage and further validation studies in similar and different populations are clearly needed before any real clinical applications. In addition, further prospective studies comparing simple versus radical hysterectomies would still be worthwhile. In this case, we believe the proposed iPMI-Power model could be applied for better and safer participant selection and recruitment. To achieve the performance benefits of the iPMI-Power, additional information on pelvic node metastasis and uterine corpus invasion is needed. The lymph node metastasis status can be evaluated by imaging including MRI, CT, PET, PET-CT, and PET-MRI or by the pathological assessment of lymph nodes obtained before surgery as a separate procedure, or during surgery with consideration of the sentinel lymph node procedure [3]. In fact, by adding only the pelvic lymph node status to the iPMI-Econ model, the predictive accuracy readily improved from 75.6% to 84.6% and the sensitivity increased from 26.7% to 56.7% with high specificity. The idea of adding uterine corpus invasion to the model is debatable and challenging. Generally, uterine corpus invasion was a histologic finding from a hysterectomy specimen and would not be detectable on clinical evaluation. Some authors suggested the possible role of pretreatment MRI in detecting uterine corpus invasion [55]. However, further studies are needed to address its accuracy. The possibility and reliability of employing transvaginal ultrasound and endometrial aspiration biopsy in assessing cervical cancer invasion to the uterine corpus should be further explored. Importantly, additional risk and expense from these extra procedures are a substantial trade-off and should be weighted carefully with the model’s predictive benefit. Furthermore, the proposed model has been developed from data retrospectively collected and stored in our division database. Inherent inaccuracy and incomplete data collection could naturally be expected. At our institution, serum biomarkers were not collected prior to the surgery for early-stage cervical cancer. Therefore, we did not have this information available for the model development. This could be considered another limitation of this study and the potential role of the biomarkers as predictors for PMI clearly deserves further evaluation. In addition, as the model is based on the single institutional data, generalizability to other population needs further exploration.

## 5. Conclusions

In this study, we proposed iPMI (i.e., iPMI-Power and iPMI-Econ), an RF-based predictor for the identification of cancer metastasis in the parametrium in patients with early-stage cervical cancer, who were typical candidates for primary radical surgery. To the best of our knowledge, the iPMI model is the first ML-based predictive model designed for the identification of PMI in early-stage cervical cancer patients. The iPMI model may accurately predict PMI in early-stage cervical cancer patients who are surgical candidates. It may provide a simpler, inexpensive, and effective method to guide important clinical decision-making. However, before the model can be implemented at the point of care, it should be further validated in larger external cohorts and updated to confirm its predictive performance in particular populations.

## Figures and Tables

**Table 1:** Comparison of clinicopathological factors between the training and the independent testing dataset.

Characteristics	Training Set (*n* = 1112)	Testing Set (*n* = 116)	*p*-Value
Age (year)	47.33 ± 8.98	48.44 ± 9.85	0.21
Parity	2.00 (1.00–2.00)	2.00 (1.00–2.00)	0.12
HIV positivity	7 (0.6)	3 (2.6)	0.03 *
Menopause	378 (34.0)	47 (40.5)	0.16
Underlying medical disease	379 (34.1)	47 (40.5)	0.17
Previous abdominal surgery	382 (34.4)	51 (44.0)	0.04 *
Prior conization	487 (43.8)	44 (37.9)	0.23
Tumor appearance			0.28
No gross lesion	489 (46.3)	46 (41.5)	
Exophytic	224 (21.2)	26 (23.4)	
Infiltrative	298 (28.2)	32 (28.8)	
Ulcerative	13 (1.2)	0 (0.0)	
Mixed	33 (3.1)	7 (6.3)	
Tumor size (cm)	1.50 (0.00–3.00)	2.00 (0.00–3.00)	0.10
Stage			0.21
IA	204 (18.3)	14 (12.0)	
IB1	779 (70.1)	88 (75.9)	
IB2	27 (2.4)	5 (4.3)	
IIA	102 (9.2)	9 (7.8)	
Final histology			0.27
Squamous	742 (66.7)	71 (61.2)	
Adenocarcinoma	256 (23.0)	36 (31.1)	
Adenosquamous	72 (6.5)	4 (3.4)	
Neuroendocrine	30 (2.7)	4 (3.4)	
Others	12 (1.1)	1 (0.9)	
Depth of invasion			0.07
Inner1/3	122 (15.5)	14 (15.1)	
Middle1/3	180 (22.9)	12 (12.9)	
Outer1/3	485 (61.6)	67 (72.0)	
Uterine metastasis			0.19
No	992 (89.2)	100 (86.2)	
Yes	95 (8.5)	15 (12.9)	
HSIL	25 (2.3)	1 (0.9)	
Vaginal metastasis			0.03 *
No	896 (80.9)	91 (78.5)	
Yes	125 (11.3)	21 (18.1)	
HSIL	87 (7.8)	4 (3.4)	
Pelvic LN metastasis	189 (17.0)	24 (20.7)	0.20
LVSI number in surgical specimen	2.00 (0.00–14.00)	3.00 (0.00–15.00)	0.23
Parametrial metastasis	171 (15.4)	30 (25.9)	<0.01 *

Data expressed as median (interquartile range) or number (%). LN; Lymph node, LVSI; Lymphovascular space invasion, HSIL; High grade squamous intraepithelial lesion * statistically significant.

**Table 2:** Independent dataset testing results of RF models with various combinations of clinicopathological factors.

Factor ^a^	Ac	Sn	Sp	MCC	AUC
baseline	0.756	0.267	0.914	0.231	0.758
	(0.678–0.834)	(0.186–0.348)	(0.863–0.965)	(0.154–0.308)	(0.680–0.836)
baseline+pelvicme	0.846	0.567	0.935	0.553	0.85
	(0.780–0.912)	(0.477–0.657)	(0.890–0.980)	(0.463–0.643)	(0.785–0.915)
baseline+utmet	0.793	0.367	0.942	0.392	0.768
	(0.719–0.867)	(0.279–0.455)	(0.899–0.985)	(0.303–0.481)	(0.691–0.845)
baseline+vgmet	0.732	0.3	0.871	0.195	0.644
	(0.651–0.813)	(0.217–0.450)	(0.810–0.932)	(0.123–0.267)	(0.557–0.731)
baseline+pelvicme+utmet	0.862	0.6	0.953	0.618	0.905
	(0.799–0.925)	(0.511–0.689)	(0.914–0.987)	(0.530–0.706)	(0.852–0.958)
baseline+pelvicme+vgmet	0.821	0.433	0.946	0.461	0.841
	(0.751–0.891)	(0.343–0.523)	(0.905–0.987)	(0.370–0.552)	(0.774–0.908)
baseline+utmet+vgmet	0.793	0.367	0.942	0.392	0.768
	(0.719–0.867)	(0.279–0.455)	(0.899–0.985)	(0.303–0.481)	(0.691–0.845)
baseline+pelvicme+utmet+vgmet	0.836	0.533	0.942	0.54	0.879
	(0.769–0.903)	(0.442–0.624)	(0.899–0.985)	(0.449–0.631)	(0.820–0.938)

^a^ baseline: preoperative clinicopathological factors, utmet: uterine metastasis, vgmet: vaginal metastasis, pelvicme: pelvic lymph node metastasis. The values are expressed with a 95% confidence interval.

**Table 3:** Independent dataset testing results of the proposed iPMI and other well-known ML-based classifiers.

Classifier ^a^	Ac	Sn	Sp	MCC	AUC
iPMI-Power	0.862	0.6	0.953	0.618	0.905
	(0.799–0.925)	(0.511–0.689)	(0.914–0.992)	(0.530–0.706)	(0.852–0.958)
SVM	0.802	0.6	0.872	0.477	0.761
	(0.729–0.875)	(0.511–0.689)	(0.811–0.933)	(0.386–0.568)	(0.683–0.839)
DT	0.819	0.6	0.895	0.513	0.751
	(0.749–0.889)	(0.511–0.689)	(0.839–0.951)	(0.422–0.604)	(0.672–0.830)
XGB	0.862	0.567	0.965	0.616	0.873
	(0.799–0.925)	(0.477–0.657)	(0.932–0.998)	(0.527–0.705)	(0.812–0.934)
kNN	0.802	0.567	0.884	0.467	0.725
	(0.729–0.875)	(0.477–0.657)	(0.826–0.942)	(0.376–0.558)	(0.644–0.806)
iPMI-Econ	0.756	0.267	0.914	0.231	0.758
	(0.678–0.834)	(0.186–0.348)	(0.863–0.965)	(0.154–0.308)	(0.680–0.836)
MLP	0.836	0.667	0.895	0.568	0.843
	(0.769–0.903)	(0.581–0.753)	(0.839–0.951)	(0.478–0.658)	(0.777–0.909)
LR	0.784	0.833	0.767	0.54	0.869
	(0.729–0.859)	(0.765–0.901)	(0.690–0.844)	(0.449–0.631)	(0.808–0.930)
NB	0.664	0.867	0.593	0.403	0.844
	(0.578–0.750)	(0.805–0.929)	(0.504–0.682)	(0.314–0.492)	(0.778–0.910)

^a^ DT: decision tree, kNN: k-nearest neighbor, LR: logistic regression, MLP: multi-layer perceptron, NB: naive Bayes, SVM: support vector machine, XGB: extreme gradient boosting. The values are expressed with a 95% confidence interval.

## Data Availability

The data presented in this study are available on request from the corresponding author.

## Supplementary Materials

The following are available online at https://www.mdpi.com/article/10.3390/diagnostics11081454/s1, Table S1: Cross-validation results of RF models with various combinations of clinicopathological factors, Table S2: Cross-validation results of the proposed iPMI and other well-known ML-based classifiers.
