# Supplementary material for: iPMI: Machine Learning-Aided Identification of Parametrial Invasion in Women with Early-Stage Cervical Cancer
Source: Diagnostics (Basel). 2021 Aug 12;11(8):1454. doi: 10.3390/diagnostics11081454 (PMC8391438; doi:10.3390/diagnostics11081454)
Supplement: Supplementary file 1 [file diagnostics-11-01454-s001.zip › diagnostics-1314688-supplementary.pdf]

Table S1 Cross-validation results of RF models with various combinations of clinicopathological factors

**Factor**[a]	**Ac**	**Sn**	**Sp**	**MCC**	**AUC**
baseline	0.882	0.872	0.894	0.767	0.950
baseline+pelvicme	0.907	0.913	0.900	0.814	0.967
baseline+utmet	0.896	0.882	0.912	0.794	0.956
baseline+vgmet	0.895	0.899	0.892	0.791	0.964
baseline+pelvicme+utmet	0.915	0.917	0.913	0.830	0.971
baseline+pelvicme+vgmet	0.918	0.915	0.922	0.837	0.973
baseline+utmet+vgmet	0.904	0.900	0.909	0.809	0.963
baseline+pelvicme+utmet+vgmet	0.918	0.918	0.919	0.838	0.977

[a]baseline: preoperative clinicopathological factors, utmet: uterine metastasis, vgmet: vaginal metastasis, pelvicme: pelvic lymph node metastasis

Table S2. Cross-validation results of the proposed iPMI and other well-known ML-based classifiers.

Classifier[a]	Ac	Sn	Sp	MCC	AUC
iPMI-Power	0.915	0.917	0.913	0.830	0.971
XGB	0.907	0.900	0.917	0.815	0.972
kNN	0.911	0.936	0.885	0.823	0.911
SVM	0.867	0.851	0.882	0.734	0.941
iPMI-Econ	0.882	0.872	0.894	0.767	0.950
DT	0.879	0.890	0.869	0.759	0.882
MLP	0.876	0.866	0.886	0.753	0.941
LR	0.779	0.792	0.767	0.560	0.841
NB	0.716	0.839	0.594	0.446	0.819

[a]DT: decision tree, kNN: k-nearest neighbor, LR: logistic regression, MLP: multi-layer perceptron, NB: naive Bayes, SVM: support vector machine, XGB: extreme gradient boosting
